# Case report: Prolonged and severe hungry bone syndrome after parathyroidectomy in X-linked hypophosphatemia

**DOI:** 10.3389/fendo.2024.1496386

**Published:** 2025-01-07

**Authors:** Giulia Puliani, Valeria Hasenmajer, Matteo Spaziani, Federico Frusone, Chiara Tarantino, Francesco Angelini, Ludovica Vincenzi, Riccardo Lubrano, Alessia Marcellino, Marco Biffoni, Andrea M. Isidori

**Affiliations:** ^1^ Oncological Endocrinology Unit, Istituto di Ricovero e Cura a Carattere Scientifico (IRCCS) Regina Elena National Cancer Institute, Rome, Italy; ^2^ Department of Experimental Medicine, Sapienza University of Rome, Rome, Italy; ^3^ Department of Theorethical and Applied Sciences, eCampus University, Novedrate, Italy; ^4^ Department of Surgical Sciences, Sapienza University of Rome, Rome, Italy; ^5^ Pediatrics and Neonatology Unit, Maternal-Child Department, Santa Maria Goretti Hospital, Sapienza University of Rome, Rome, Italy; ^6^ Centre for Rare Diseases (Endo-ERN accredited), Policlinico Umberto I, Rome, Italy

**Keywords:** tertiary hyperparathyroidism, hungry bone syndrome, X-linked hypophosphatemia, burosumab, FGF23, parathyroidectomy

## Abstract

Tertiary hyperparathyroidism is characterized by hypercalcemia resulting from autonomous parathyroid hormone production and usually occurs after a prolonged period of secondary hyperparathyroidism. This condition can be a complication of X-linked hypophosphatemia (XLH), a rare genetic disease characterized by renal phosphate loss and consequent hypophosphatemia. Parathyroidectomy is considered the first-line therapy but surgical intervention can be complicated by hungry bone syndrome. A male Caucasian patient presented with XLH, diagnosed at the age of 3 years. At the age of 21, tertiary hyperparathyroidism occurred. Neck ultrasonography, neck magnetic resonance imaging, and ^99^Tc-sestamibi parathyroid scintigraphy revealed two hyperplastic parathyroid glands. To minimize the risk of hypercalcemia, calcimimetic therapy was initiated. After 6 months and preparation with 1,25-dihydroxy vitamin D, the patient underwent total parathyroidectomy with autotransplantation of half of a parathyroid gland into the sternocleidomastoid muscle. Histopathological examination revealed diffuse microscopical hyperplasia of the parathyroid glands. Despite oral supplementation with calcium carbonate and calcitriol, severe hypocalcemia developed on the second postoperative day, attributable to hungry bone syndrome. This finding was confirmed by an increase in bone turnover markers and a reduction in urinary calcium excretion. Hypocalcemia correction required continuous infusion of calcium gluconate for over 2 months. After approval, the patient began burosumab therapy with significant benefits. This case illustrates the complexity of treating tertiary hyperparathyroidism and mineral metabolism in patients with XLH. The hungry bone syndrome can complicate parathyroidectomy, exposing the patients to life-threatening risks. Burosumab therapy may reduce the risk of tertiary hyperparathyroidism developing in these patients.

## 1 Introduction

Tertiary hyperparathyroidism is a complication of prolonged secondary hyperparathyroidism and represents a state of autonomous parathyroid tissue function characterized by hypercalcemic hyperparathyroidism (1). Causes of tertiary hyperparathyroidism include chronic kidney disease, prolonged osteomalacia due to vitamin d deficiency and X-linked hypophosphatemia (XLH) (2–4), which is the most common cause of hereditary rickets caused by a mutation in the phosphate regulating endopeptidase homolog X-linked (PHEX) gene (5). This mutation results in high fibroblast growth factor 23 (FGF23) levels, leading to increased renal phosphate excretion, renal 1-alpha-hydroxylase downregulation, and hypophosphatemia (6, 7). Clinical management of XLH is burdened by several complications, with hyperparathyroidism affecting up to 83.3% of patients (8, 9). Tertiary hyperparathyroidism usually requires parathyroidectomy (10). However, data on the long-term efficacy of parathyroidectomy in patients with XLH and hypercalcemic hyperparathyroidism are still scarce and limited to case series (9, 11, 12), which have described high recurrence rates (8). A relatively uncommon but serious adverse effect of parathyroidectomy is hungry bone syndrome (HBS), defined as severe and prolonged (lasting longer than the fourth postoperative day) hypocalcemia (13). This condition can occur after parathyroidectomy for severe hyperparathyroidism (14) but few data are available as a consequence of tertiary hyperparathyroidism due to XLH (11). Our report aims to describe the challenges of tertiary hyperparathyroidism management in XLH and its complications, also through the collection and discussion of the available evidence, often described singularly, also mutuated from similar diseases (such as tertiary hyperparathyroidism due to other diseases), to provide a support for the challenges experienced by the clinicians approaching a rare complication of a rare disease, as XLH.

## 2 Case report

A 21-year-old man presented with hypercalcemia and hyperparathyroidism. Considering clinical examination and biochemical evaluation showing hypophosphatemia and increased renal phosphate excretion, the patient was diagnosed with XLH at the age of 3 years and was undergoing conventional, integrative therapy with 125 mg of phosphorus, four times a day. This dosage was slightly lower than current XLH guidelines, which prescribe a daily phosphate dose of 20-60 mg/kg. However, it still enabled the patient to manage his symptoms, maintained serum fasting phosphoremia within the normal range for age, promoted optimal growth, and prevented bone pain or fractures. Moreover, active vitamin D supplementation (1,25-dihydroxy vitamin D) was prescribed until the age of 9. During follow-up, secondary hyperparathyroidism was documented on several occasions. The patient had a familial history of myocardial infarction, hypertension, and pulmonary carcinoma.

**Figure 1:**
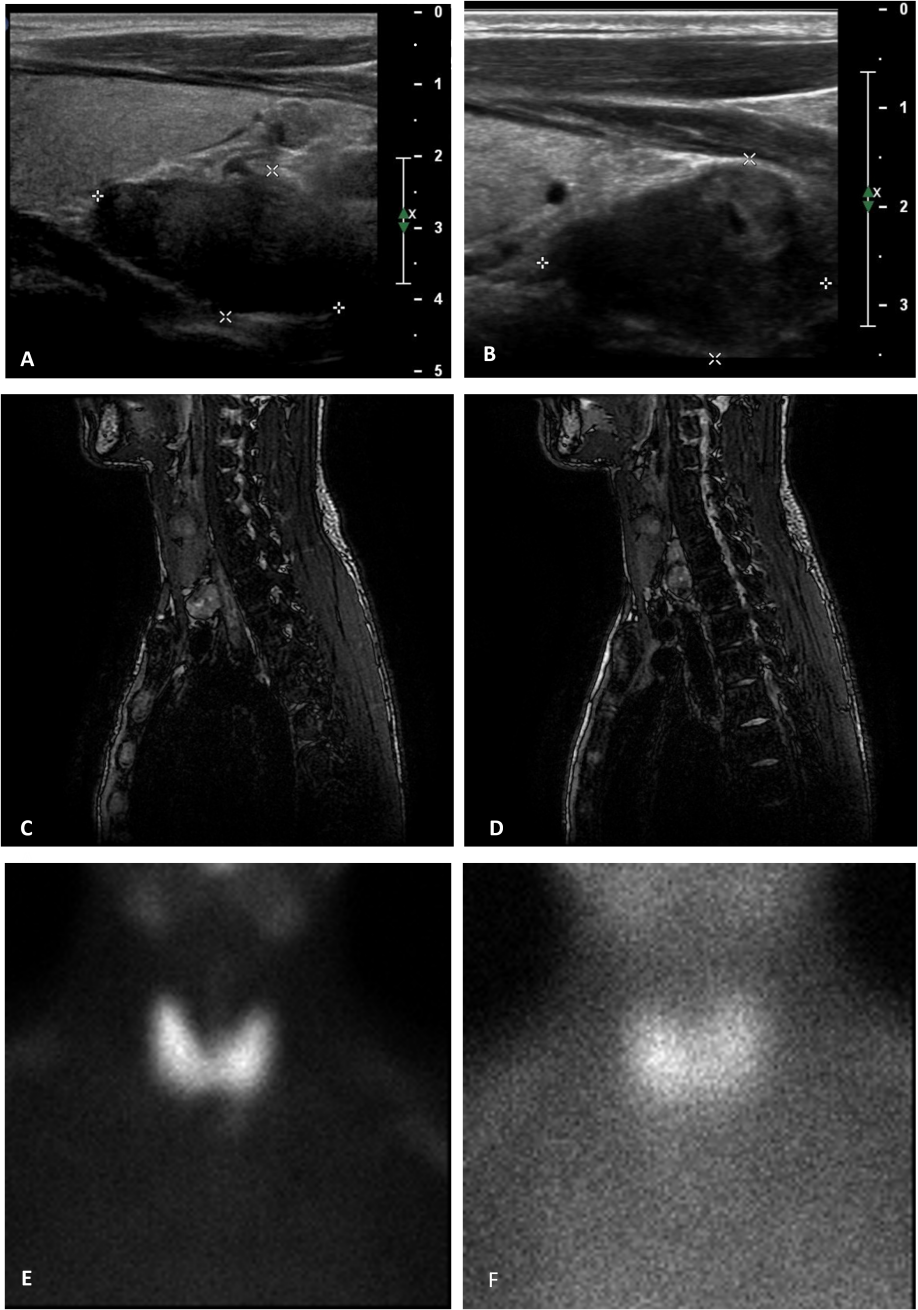
Summary of the patient's pre-operative examinations. **(A, B)** neck ultrasonography. Hyperplastic parathyroids are located below the level of the thyroid gland on the right **(A)** and left **(B)** sides of the neck (maximum diameter of 2.5 and 3.0 cm, respectively). **(C, D)** magnetic resonance Imaging. In the opposition phase, T2-weighted magnetic resonance images of the neck show hyperplastic parathyroid glands located under the left thyroid lobe **(A)** and behind the right thyroid lobe **(B)**, characterized by hyperintensity signal, measuring 2.0 × 1.2 × 3.5 cm and 2.3 × 1.3 × 2.2 cm, respectively (APD × TD × LD). **(E, F)** parathyroid scintigraphy (99mTc 174 MBq + 99mTc-MIBI 326MBq). Pictures show an area of residual ^99m^Tc-MIBI fixation located near the left thyroid lobe, compatible with a hyperfunctional parathyroid.

On the first presentation, the patient’s height and weight were 178 cm and 83 kg, respectively. Signs of hypercalcemic hyperparathyroidism (PTH, 1,009 pg/mL; reference range, 5–65 pg/mL), with a total serum calcium level of 3.24 (reference range, 2.12–2.50) mmol/L, were noted. Neck ultrasonography revealed two hypoechoic nodules under the right and left thyroid lobes, measuring 2.5 and 3 cm, respectively. These findings were compatible with those of hyperplastic parathyroid glands, which was confirmed by neck magnetic resonance imaging that revealed the presence of two hyperintense nodules in the T2-weighted sequences. ^99^Tc-Sestamibi parathyroid scintigraphy confirmed that the left nodule was compatible with hyperfunctioning parathyroid. Diagnostic examinations are reported in **Figure 1**. Considering the patient’s underlying condition and history of secondary hyperparathyroidism, the diagnosis of tertiary hyperparathyroidism was confirmed. The first-line therapy for this condition is total parathyroidectomy (1). Given the severely high serum calcium levels, calcimimetic therapy was initiated before surgery to minimize pre- and intraoperative risks. In the 3 weeks before surgery, as a preventive measure against a possible HBS, the patient also took calcitriol supplementation of 0.5 μg/day. With an initial dose of 60 mg of cinacalcet, the patient achieved a reduction in serum calcium levels. However, after 2 months of continuous treatment, the patient required incremental doses up to 120 mg daily. After 6 months of therapy hypercalcemia recurred, along with an increase in the right nodule volume (maximum diameter 3.7 cm *vs* 2.5 cm). Therefore, the patient underwent total parathyroidectomy with autotransplantation of half of a parathyroid gland into the sternocleidomastoid muscle. Intraoperative PTH sampling was performed to ensure the complete removal of the hyperfunctioning parathyroid tissue, with basal and postoperative PTH levels measuring 1,821 and 98.3 pg/mL, respectively. Histopathological examination revealed diffuse microscopic hyperplasia of the 3 excised parathyroids; for the reimplantation the pathologist chose the parathyroid with less microscopic hyperplasia or other alteration.

The postoperative therapeutic regimen included the following: oral calcium carbonate supplementation, 1,000 mg (three times daily); oral calcitriol, 0.5 μg (three times daily); intravenous calcium gluconate supplementation, 2.1 mEq (four times daily); magnesium pidolate, 1.5 g (three times daily); and half of the previous phosphorus supplementation (125 mg, twice daily). Nevertheless, severe and prolonged hypocalcemia developed (serum calcium, nadir 5.4 mg/dL), which was associated with digital and perioral paresthesia and a positive Chvostek’s sign, without any prolongation of the QT interval on electrocardiography. After surgery, the diagnosis of post-operative hypoparathyroidism was excluded due to the other laboratory values, including hypocalciuria (14.4 mg/24h) and increased levels of osteocalcin, c-terminal telopeptide of type I collagen (CTX) and alkaline phosphatase, that were consistent with HBS. Serum phosphoremia remained within the normal range after parathyroidectomy.

**Figure 2:**
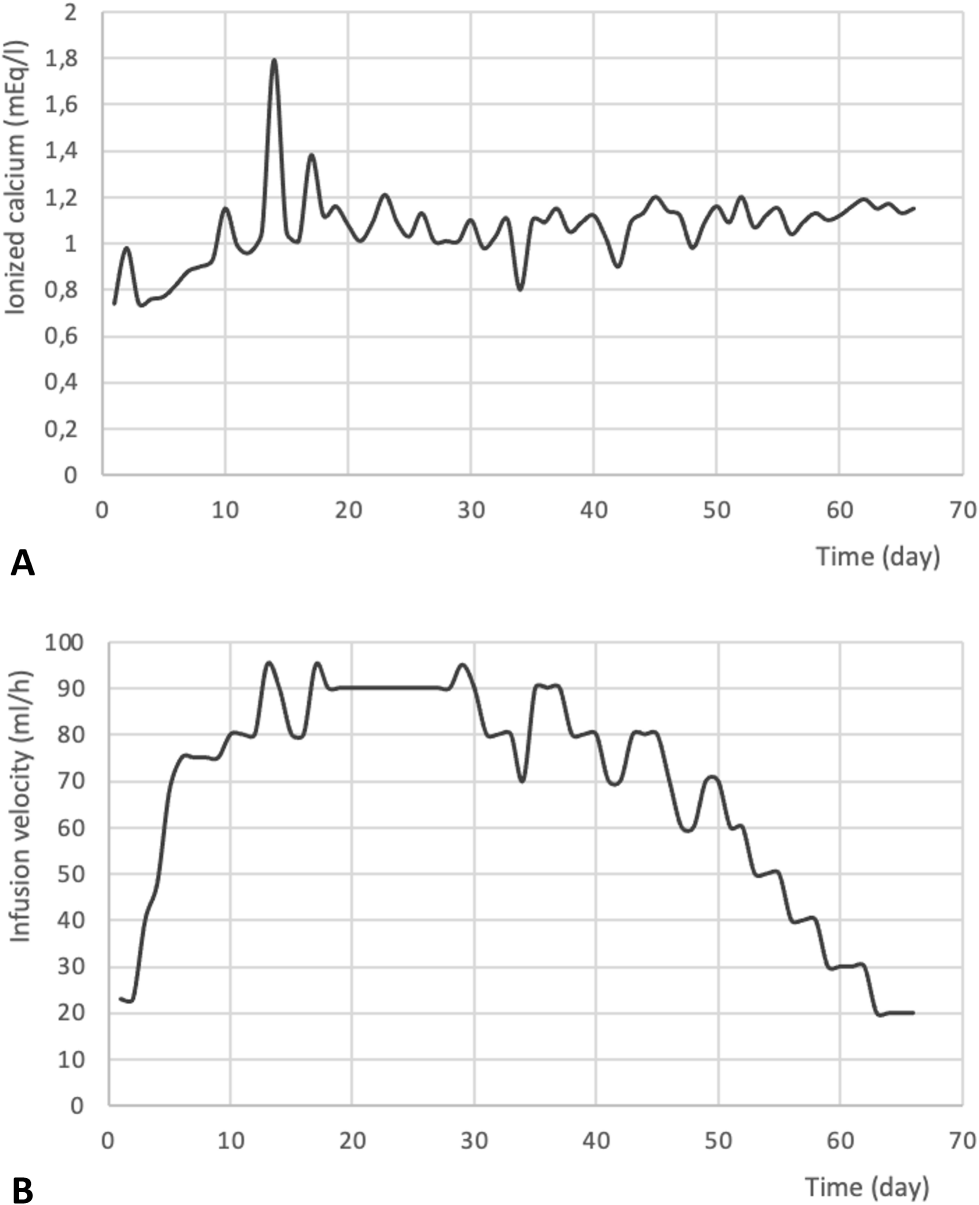
Trend of postoperative ionized calcium levels (mEq/l) **(A)** and calcium gluconate infusion velocity (ml/l) **(B)**. to acknowledge the contributions of specific colleagues, institutions, or agencies that aided the efforts of the authors.

The patient required continuous infusion of calcium gluconate (12.6 mEq in 500 ml of saline solution), with the infusion rate adjusted based on serum calcium levels (**Figure 2**). He also required oral calcitriol (1 μg, three times daily) and magnesium sulfate (20 mEq, three times daily) supplementation. Hypocalcemia correction required nearly two months of continuous calcium gluconate infusion during the hospital stay.

Bone formation markers reduced progressively during hospitalization, and urinary calcium excretion increased. A PTH level of 28 pg/mL on the 20th postoperative day demonstrated the only partial recovery of PTH function with parathyroid autotransplantation (**Table 1**). Unfortunately, FGF-23 dosage was unavailable in our hospital.

**Table 1:** Postoperative bone metabolism biochemical parameters.

	Range	Intraop	Day 1	Day 10	Day 20	Day 30	Day 40	Day 60
ALP (UI/l)	40–129	–	388	357	277	242	152	136
24-h calciuria (mg/24 h)	100–320	–	14.4	44.0	89.1	65.0	86.3	102.4
Osteocalcin (ng/mL)	24–70	–	282.0	403.5	289.1	262.9	162.0	92.0
CTX (ng/mL)	0.05–0.75	–	0.89	0.98	0.52	0.24	0.19	0.22
PTH (pg/mL)	15–65	98.3	5	12	28	26	29	32

ALP, alkaline phosphatase; CTX, C-terminal telopeptide of type I collagen; intraop, intraoperative.

The patient was discharged 60 days after parathyroidectomy with the following treatment: oral calcitriol 0.5 µg (three times daily), calcium carbonate 1,000 mg (twice daily), and phosphorus 125 mg (twice daily).

In the subsequent follow-ups, calcium levels consistently remained within the normal range, allowing for a gradual reduction and discontinuation of calcium supplementation. Six months after the surgery, the patient was only receiving a daily dose of 0.5 µg of calcitriol together with the supplemental dose of 125 mg of phosphorus, administered in two daily doses.

The dual-energy X-ray absorptiometry scan, performed 6 months after surgery, revealed an increase in bone mineral density (BMD) as evaluated by the lumbar Z-score, which changed from +1.8 to +3.8. That change was consistent with rapid mineralization after PTH normalization.

Despite the minimal therapeutic dosage and normal calcium and phosphorus levels, severe nephrocalcinosis occurred two years after surgery, followed by a significant increase in PTH levels (approximately 200 pg/mL). For secondary hyperparathyroidism, nephrocalcinosis, and patient’s symptoms, after the Food and Drug Administration (FDA) approval in 2018, approximately 3 years after surgery, burosumab therapy was initiated at a monthly single dose of 90 mg (1 mg/kg of body weight) subcutaneously. As per the drug’s technical sheet, the patient discontinued the supplementary phosphate therapy 1 week before the first injection. Moreover, to access this therapy, the patient previously underwent a genetic test that confirmed the diagnosis of XLH, revealing a hemizygous mutation in the *PHEX* gene (c.1735G>A [p.Gly579Arg]). In Italy, treatment with burosumab must be preceded by a withdrawal of phosphate supplementation; in this phase, immediately before burosumab starting, the patient had low phosphorus levels (0.32 mmol/L), accompanied by severe symptoms including headache, cramps, muscle pain, and weakness. Such symptoms rapidly resolved after initiating the new medication, leading to a significant improvement in the serum parameters (**Table 2**) and the patient’s quality of life. Such symptoms rapidly resolved after initiating the new medication, leading to a significant improvement in serum parameters (**Table 2**) and the patient’s quality of life.

**Table 2:** Biochemical parameter changes before and 3 months after the start of burosumab therapy.

	Before burosumab	After 3 months of burosumab
P (mmol/L)	0.33	0.9
PTH (pg/mL)	200	109
TRP (%)	56	84

P, phosphorus; TRP, tubular reabsorption of phosphate.

## 3 Discussion

We describe the clinical case of a patient with XLH complicated by tertiary hyperparathyroidism who underwent surgical intervention complicated by severe HBS.

Longstanding chronic kidney disease is the most common cause of tertiary hyperparathyroidism. However, tertiary hyperparathyroidism is also frequently observed in XLH, a hereditary metabolic bone syndrome characterized by renal phosphate wasting and inappropriately low levels of calcitriol. This condition leads to hypophosphatemia and abnormal bone mineralization and was first described by Albright in 1937 (15). Conventional therapy involves oral supplementation with high doses of phosphate (16). However, the temporary hyperphosphatemia resulting from this treatment decreases ionized calcium levels and the production of 1,25-dihydroxyvitamin D. This stimulates PTH secretion, leading to secondary hyperparathyroidism. Nevertheless, secondary hyperparathyroidism has also been described in patients with untreated XLH, which is likely related to 1,25-dihydroxyvitamin D deficiency attributable to FGF23 excess (17). Long-term secondary hyperparathyroidism can affect the autonomous functioning of the parathyroid glands, resulting in hypercalcemia (tertiary hyperparathyroidism) (8, 9, 18). In patients with XLH, the prevalence of hypercalcemic hyperparathyroidism (tertiary hyperparathyroidism) is between 10% (6) and 16% (8). No guidelines are available for the treatment of tertiary hyperparathyroidism, but clinical management can be guided by available studies (1, 19). The current indications for parathyroidectomy include: symptomatic, persistent, or significantly increased (> 2.74 mmol/L) hypercalcemia; high parathyroid hormone levels; hypophosphatemia; decreased BMD; kidney function decline associated with hyperparathyroidism (20). Surgical treatment aims to reduce the parathyroid mass and cell number, thereby normalizing the serum calcium concentration.

The main surgical procedures for tertiary hyperparathyroidism are subtotal parathyroidectomy, in which only one or two parathyroid tissues are removed, and total parathyroidectomy, which involves removing all parathyroid tissues. Total parathyroidectomy can eventually be associated with the autotransplantation of 1-2 normal parathyroids in the forearm or neck muscles (21). Some authors assert that total parathyroidectomy without autotransplantation can be protective against recurrent hypercalcemia during follow-up, and that permanent hypoparathyroidism can be successfully managed with lifelong oral supplementation of calcitriol (and calcium supplementation, when necessary) with minimal risk of developing adynamic bone disease (22) or by 1-34 and 1-84 PTH replacement therapy, for reducing the risk of conventional treatment supplementation (23). In addition, the absence of PTH can be protective for hypophosphatemia, since it has been demonstrated that PTH is essential for the phosphaturic effect of FGF-23 (24, 25) (Other authors argue that subtotal parathyroidectomy is the preferred surgical treatment because it reduces the risk of hypocalcemia, with no significant differences in operative time, length of hospital stays, gland weight, or other laboratory parameters (26, 27). Intraoperatively, the serum PTH level must be measured (28). In most cases of subtotal parathyroidectomy, 10-15 min after the excision, the serum PTH level should decrease by >50% from the initial baseline value (29). Conversely, total parathyroidectomy is considered adequate if the immediate postoperative serum PTH levels are <2 pg/mL (22). In our patient, despite the initial postoperative PTH level of 98.3 pg/ml, which was indicative of therapeutic success, considering the preoperative PTH value of 1,821 pg/mL, the positive outcome of the surgical procedure was further, albeit paradoxically, demonstrated by the emergence of the HBS.

Only a few studies have evaluated the best surgical procedure for XLH-associated tertiary hyperparathyroidism. A case series reported recurrence of hypercalcemia after parathyroidectomy in six out of the eight patients in one cohort of patients with XLH (7) after a median of 6 years, whereas in another cohort, patients showed normal postoperative calcium and PTH levels; however, the follow-up duration was shorter (median of 44 months) (6). No clear guidelines are available for parathyroid surgery in hypercalcemic hyperparathyroidism in hypophosphatemic rickets, and the number of parathyroid glands removed varies among patients. However, parathyroid multiple-gland hyperplasia is expected, making it necessary to inspect all parathyroid tissue during surgery (11). No study compared the risk of HBS after parathyroidectomy for tertiary hyperparathyroidism due to XLH or other etiology. In addition, the risk of HBS after parathyroidectomy is high in a reported case series (4/5 patients, 80%) (11), but the low number of patients described impedes drawing a definite conclusion on this topic. Considering this, our patient, who had significantly high PTH and calcium levels, underwent the removal of all parathyroid glands and autotransplantation of half of a parathyroid gland into the sternocleidomastoid muscle. Despite avoiding autotransplantation could restore phosphate levels blocking the phosphaturic effect of FGF-23, we decided to prevent permanent and severe hypoparathyroidism in our patient, considering it is a life-threatening condition with a great impact on bone metabolism and quality of life (30, 31). Considering the actual role of burosumab for patients with XLH, we believe that permanent complications of permanent hypoparathyroidism should be avoided.

Alternative treatment options include calcimimetics, such as cinacalcet, which inhibits PTH secretion by modulating the calcium-sensing receptor in the parathyroid gland. Although calcimimetics are not officially approved for the treatment of tertiary hyperparathyroidism, a few clinical trials have reported that these drugs can reduce or normalize calcium and PTH levels without changes in renal function or major adverse events (32, 33). A recent review compared the efficacy and side effects of surgery and medical therapy for tertiary hyperparathyroidism caused by renal failure. Parathyroidectomy for tertiary hyperparathyroidism has higher cure rates than cinacalcet therapy, with only mild side effects and complications associated with both treatment modalities (34). Treatment with cinacalcet has been described in a few patients with XLH. DeLacey et al. reported that calcimimetics therapy was attempted in 35% of patients, which yielded variable results (8). Despite the general short-term safety of cinacalcet therapy, most patients ultimately underwent parathyroidectomy because of progressive renal failure, side effects, worsening of biochemical control, or lack of efficacy in normalizing serum calcium levels. In one case report, calcimimetics therapy was well tolerated, resulting in sustained calcium and PTH level normalization for 6 months (35). Another described how cinacalcet successfully treated XLH for 3 years (36). To lower the surgical risk and considering the long waiting list for surgical procedures, our patient was initially pretreated with cinacalcet to reduce calcium levels. Notably, the complexity of the clinical picture, combined with the limited therapeutic success, required increasing the dosage of cinacalcet from 60 to 120 mg and, finally, the need for surgical intervention.

A significant decrease in serum calcium levels is usually observed after parathyroidectomy in primary hyperparathyroidism. However, the rapid reduction in bone resorption can result in severe and prolonged hypocalcemia with low or normal levels of phosphate, which is termed HBS (14). Its incidence varies from 13% in earlier case series to 24%–87% in more recent Asian studies, as reported in a relatively recent systematic literature review (14). HBS pathogenesis is not entirely understood. In patients with hyperparathyroidism, the preoperative bone turnover rate is likely to be high. After the PTH levels decrease, the reduction in osteoclastic activity results in decreased bone remodeling and increased bone mass (8, 14). The increase in bone formation explains the profound drop in serum calcium, phosphate, and magnesium levels. Other contributing factors include functional or relative hypoparathyroidism and reduced intestinal calcium absorption caused by a decrease in 1-25 dihydroxycholecalciferol levels. After surgical intervention, there was a marked increase in remineralization, more pronounced compared to patients with other forms of hyperparathyroidism, such as secondary and tertiary hyperparathyroidism in kidney disease (37, 38). One possible explanation is, besides biological differences, the young age of the patient at surgical intervention, probably before the reaching of the peak bone mass.

Some studies have attempted to identify risk factors for HBS development. Latus et al. evaluated 84 patients who underwent parathyroidectomy and found that HBS developed in 43 patients (51%) after surgery. Lower preoperative calcium levels and younger age at the time of surgery were significant predictors of HBS (39). Whether young age can be a risk factor for HBS is unclear; the authors hypothesized that the increases in bone formation and osteoblast activity after parathyroidectomy are more pronounced in younger patients than in older ones. Lo-Yi Ho et al. focused on secondary hyperparathyroidism in patients undergoing dialysis, and sex (male), younger age, body weight, and serum preoperative alkaline phosphatase and calcium levels were found to be predictors of HBS. The preoperative use of active vitamin D analogs had no significant effect on HBS development (40).

In this context, two studies aiming to create a predictive risk system to develop HBS in patients with renal hyperparathyroidism have been recently published (41, 42). Ramesh et al. observed that elevated preoperative serum PTH and ALP levels are identified as significant predictors, leading to a two-point scoring system with 96.8% diagnostic accuracy (41). Our case report supports the validity of this risk system also in XLH: our patient presented with preoperative values of PTH higher than 1000 pg/ml and alkaline phosphatase higher than 150 U/L) and developed HBS. Otherwise, Amjad et al. established for patients with end-stage renal disease undergoing parathyroidectomy for secondary hyperparathyroidism a risk score based on factors such as age, dialysis duration, and Elixhauser score, which effectively stratified HBS risk, ranging from 8% to 44%. The tool provides a poor positive predictive value (20.3%) but an excellent negative predictive value (89.3%) (42).

Regarding potential pharmacological therapy to prevent HBS, the literature offers conflicting data. Some authors have suggested that preoperative calcitriol therapy could prevent HBS development after surgery (43). However, Heath demonstrated in the development of severe hypocalcemia after parathyroidectomy no difference between the calcitriol-treated and untreated groups (44).

A recent study retrospectively evaluated 19 patients who underwent parathyroidectomy (45). Among the 11 patients treated with zoledronic acid preoperatively, none developed HBS, whereas HBS developed in three of the eight patients without pretreatment. Similarly, Lee et al. demonstrated that none of the six patients treated with clodronate or pamidronate had postoperative complications, whereas hypocalcemia developed in 9 out of the 17 without treatment (46). In a case series of 46 patients with severe bone disease, the retrospective evaluation revealed that HBS developed in only 4% of patients who received zoledronate (47).

Conversely, some case reports have shown that various bisphosphonates (including intravenous administration of pamidronate) (48) and zoledronic acid (49) were not effective in preventing HBS in patients with hyperparathyroidism. In a case report of parathyroid carcinoma, intravenous administration of pamidronate (90 mg intravenous twice) failed to prevent HBS after surgery (50). A recent meta-analysis that only included two studies supported the protective role of bisphosphonates for postoperative HBP in patients undergoing parathyroidectomy for primary hyperparathyroidism (risk ratio, 0.12; 95% CI, 0.02–0.89, I^2^ = 0%) (51).

In our case, the patient’s calcium levels and the rapid growth of the parathyroid glands, as demonstrated by neck ultrasonography, led us to choose total parathyroidectomy as the best treatment strategy. The risk of HBS development was high because of the long history of hyperparathyroidism (nearly 8 years), phosphate therapy, and young age. To avoid possible complications of hypercalcemia (calcium levels also reached 3.24 mmol/L), as mentioned earlier, the patient was treated with cinacalcet and, 3 weeks before surgery, oral calcitriol. We chose calcitriol instead of bisphosphonate in the preoperative management because of conflicting data regarding HBS prevention with bisphosphonates and the risk of poor BMD increase after parathyroidectomy associated with this class of medication (52). Despite calcitriol therapy, our patient developed HBS. In this case, longstanding hyperparathyroidism and XLH created an enhanced bone turnover in this patient, as evidenced by a significant gain in bone mass after surgery, which may have impeded HBS prevention. In addition, considering inappropriately normal levels of PTH during the profound hypocalcemia, it is not possible to exclude that concomitant transient hypoparathyroidism could have played a role in the long-standing hypocalcemia experienced by our patient. However, it is noteworthy that HBS best explains the low phosphate level. The possibility of multiple contemporary causes of hypoparathyroidism in the postoperative setting of patients affected by tertiary hyperparathyroidism for XLH should be considered in patients’ management.

Only 3 years after the parathyroid gland surgery for tertiary hyperparathyroidism was burosumab therapy initiated [the FDA approved it in 2018] (53). Burosumab is an antibody that inhibits the action of FGF23, which is responsible for phosphate renal leakage in XLH. This treatment significantly improved the patient’s quality of life, reducing symptoms and freeing him from conventional daily therapy (54). Despite the limited body of research that comprehensively assessed the effect of burosumab on adults, as well-summarized in a recent review (55), a single-arm, open-label study focused on long-term safety and effectiveness by evaluating biological markers, pain levels, and functional ability scores. Notably, this study demonstrated the ability of burosumab to significantly reduce circulating PTH levels by week 72 of treatment (56), echoing our patient’s experience (already evident after 3 months of treatment). Although further research on burosumab therapy in adults is warranted, this innovative treatment is expected to protect patients with XLH against a range of complications, including tertiary hyperparathyroidism.

## 4 Conclusions

This clinical case unravels the complex treatment of tertiary hyperparathyroidism in patients with XLH, shedding light on the absence of clear preoperative management guidelines. Although parathyroidectomy is an essential step, it can frequently be fraught with the potential for HBS, which, in turn, may prolong the hospitalization. All these conditions require a multidisciplinary approach, including endocrinologists, nephrologists, and surgeons. Burosumab therapy holds great promise in reducing the incidence of this and other side effects in patients with XLH, improving the quality of life of treated patients.

## Data Availability

The raw data supporting the conclusions of this article will be made available by the authors, without undue reservation.

## References

[1] Palumbo VD Palumbo VD Damiano G Messina M Fazzotta S Lo Monte G . Tertiary hyperparathyroidism: a review. Clin Ter. (2021) 172:241–6. doi: 10.7417/CT.2021.2322 33956045

[2] Giannini S Bianchi ML Rendina D Massoletti P Lazzerini D Brandi ML . Burden of disease and clinical targets in adult patients with X-linked hypophosphatemia. A Compr review Osteoporos. Int. (2021) 32:1937–49. doi: 10.1007/s00198-021-05997-1 PMC8510985 34009447

[3] Fraser WD . Hyperparathyroidism. Lancet. (2009) 374:145–58. doi: 10.1016/S0140-6736(09)60507-9 19595349

[4] Seshadri MS Qurttom MA Sivanandan R Shihab-al-Mohannadi Samiaman . Tertiary hyperparathyroidism in nutritional osteomalacia. Postgrad Med J. (1994) 70:595–6. doi: 10.1136/pgmj.70.826.595-b PMC2397703 7937459

[5] Alizadeh Naderi AS Reilly RF . Hereditary disorders of renal phosphate wasting. Nat Rev Nephrol. (2010) 6:657–65. doi: 10.1038/nrneph.2010.121 20924400

[6] Lecoq A-L Brandi ML Linglart A Kamenický P . Management of X-linked hypophosphatemia in adults. Metabolism. (2020) 103S:154049. doi: 10.1016/j.metabol.2019.154049 31863781

[7] Chesher D Oddy M Darbar U Sayal P Casey A Ryan A . Outcome of adult patients with X-linked hypophosphatemia caused by PHEX gene mutations. J Inherit. Metab Dis. (2018) 41:865–76. doi: 10.1007/s10545-018-0147-6 PMC6133187 29460029

[8] DeLacey S Liu Z Broyles A El-Azab SA Guandique CF James BC . Hyperparathyroidism and parathyroidectomy in X-linked hypophosphatemia patients. Bone. (2019) 127:386–92. doi: 10.1016/j.bone.2019.06.025 PMC6836672 31276850

[9] Lecoq A-L Chaumet-Riffaud P Blanchard A Dupeux M Rothenbuhler A Lambert B . Hyperparathyroidism in patients with X-linked hypophosphatemia. J Bone Miner. Res. (2020) 35:1263–73. doi: 10.1002/jbmr.3992 32101626

[10] Jamal SA Miller PD . Secondary and tertiary hyperparathyroidism. J Clin Densitom. (2013) 16:64–8. doi: 10.1016/j.jocd.2012.11.012 23267748

[11] Savio RM Gosnell JE Posen S Reeve TS Delbridge LW . Parathyroidectomy for tertiary hyperparathyroidism associated with X-linked dominant hypophosphatemic rickets. Arch Surg. (2004) 139:218–22. doi: 10.1001/archsurg.139.2.218 14769584

[12] Neal MD Deslouches B Ogilvie J . The use of pre-operative imaging and intraoperative parathyroid hormone level to guide surgical management of tertiary hyperparathyroidism from X-linked hypophosphatemic rickets: a case report. cases J. (2009) 2:7572. doi: 10.4076/1757-1626-2-7572 19918472 PMC2769362

[13] Jain N Reilly RF . Hungry bone syndrome. Curr Opin Nephrol. Hypertens. (2017) 26:250–5. doi: 10.1097/MNH.0000000000000327 28375869

[14] Witteveen JE van Thiel S Romijn JA Hamdy NAT . Hungry bone syndrome: still a challenge in the post-operative management of primary hyperparathyroidism: a systematic review of the literature. Eur J Endocrinol. (2013) 168:R45–53. doi: 10.1530/EJE-12-0528 23152439

[15] Albright F . Rickets resistant to vitamin d therapy. Arch Pediatr Adolesc. Med. (1937) 54:529. doi: 10.1001/archpedi.1937.01980030073005

[16] Haffner D Emma F Eastwood DM Duplan MB Bacchetta J Schnabel D . Clinical practice recommendations for the diagnosis and management of X-linked hypophosphataemia. Nat Rev Nephrol. (2019) 15:435–55. doi: 10.1038/s41581-019-0152-5 PMC7136170 31068690

[17] Carpenter TO Olear EA Zhang JH Ellis BK Simpson CA Cheng D . Effect of paricalcitol on circulating parathyroid hormone in X-linked hypophosphatemia: a randomized, double-blind, placebo-controlled study. J Clin Endocrinol Metab. (2014) 99:3103–11. doi: 10.1210/jc.2014-2017 PMC4154090 25029424

[18] Rivkees SA el-Hajj-Fuleihan G Brown EM Crawford JD . Tertiary hyperparathyroidism during high phosphate therapy of familial hypophosphatemic rickets. J Clin Endocrinol Metab. (1992) 75:1514–8. doi: 10.1210/jcem.75.6.1464657 1464657

[19] Parangi S Gartland RM . A call for multidisciplinary consensus guidelines for the management of tertiary hyperparathyroidism. Ann Surg. (2021) 273:e123. doi: 10.1097/SLA.0000000000004694 33378305

[20] Tang JA Friedman J Hwang MS Salapatas AM Bonzelaar LB Friedman M . Parathyroidectomy for tertiary hyperparathyroidism: A systematic review. Am J Otolaryngol. (2017) 38:630–5. doi: 10.1016/j.amjoto.2017.06.009 28735762

[21] D’Alessandro AM Melzer JS Pirsch JD Sollinger HW Kalayoglu M Vernon WB . Tertiary hyperparathyroidism after renal transplantation: operative indications. Surgery. (1989) 106:1049–55;discussion 1055–6. 2588112

[22] Sadideen HM Taylor JD Goldsmith DJ . Total parathyroidectomy without autotransplantation after renal transplantation for tertiary hyperparathyroidism: long-term follow-up. Int Urol. Nephrol. (2012) 44:275–81. doi: 10.1007/s11255-011-0069-9 21997202

[23] Puliani G Hasenmajer V Simonelli I Sada V Pofi R Minnetti M . Safety and efficacy of PTH 1-34 and 1-84 therapy in chronic hypoparathyroidism: A meta-analysis of prospective trials. J Bone Miner. Res. (2022) 37:1233–50. doi: 10.1002/jbmr.4566 PMC9545848 35485213

[24] Bai X Miao D Goltzman D Karaplis AC . Early lethality in Hyp mice with targeted deletion of Pth gene. Endocrinology. (2007) 148:4974–83. doi: 10.1210/en.2007-0243 17615144

[25] Bhadada SK Palnitkar S Qiu S Parikh N Talpos GB Rao SD . Deliberate total parathyroidectomy: a potentially novel therapy for tumor-induced hypophosphatemic osteomalacia. J Clin Endocrinol Metab. (2013) 98:4273–8. doi: 10.1210/jc.2013-2705 23956343

[26] Choi HR Aboueisha MA Attia AS Omar M ELnahla A Toraih EA . Outcomes of subtotal parathyroidectomy versus total parathyroidectomy with autotransplantation for tertiary hyperparathyroidism: multi-institutional study. Ann Surg. (2021) 274:674–9. doi: 10.1097/SLA.0000000000005059 34506323

[27] Hsieh T-M Sun C-K Chen Y-T Chou F-F . Total parathyroidectomy versus subtotal parathyroidectomy in the treatment of tertiary hyperparathyroidism. Am Surg. (2012) 78:600–6. doi: 10.1177/000313481207800544 22546135

[28] Ermer JP Kelz RR Fraker DL Wachtel H . Intraoperative parathyroid hormone monitoring in parathyroidectomy for tertiary hyperparathyroidism. J Surg Res. (2019) 244:77–83. doi: 10.1016/j.jss.2019.06.020 31279997 PMC7179078

[29] Kao PC van Heerden JA Taylor RL . Intraoperative monitoring of parathyroid procedures by a 15-minute parathyroid hormone immunochemiluminometric assay. Mayo Clin Proc. (1994) 69:532–7. doi: 10.1016/s0025-6196(12)62243-5 8189758

[30] Kontogeorgos G Mamasoula Z Krantz E Trimpou P Landin-Wilhelmsen K Laine CM . Low health-related quality of life in hypoparathyroidism and need for PTH analog. Endocr Connect. (2022) 11. doi: 10.1530/EC-21-0379 PMC8789022 34825891

[31] Khan AA Bilezikian JP Brandi ML Clarke BL Gittoes NJ Pasieka JL . Evaluation and management of hypoparathyroidism summary statement and guidelines from the second international workshop. J Bone Miner Res. (2022) 37:2568–85. doi: 10.1002/jbmr.4691 36054621

[32] Serra AL Savoca R Huber AR Hepp U Delsignore A Hersberger M . Effective control of persistent hyperparathyroidism with cinacalcet in renal allograft recipients. Nephrol. Dial. Transplant. (2007) 22:577–83. doi: 10.1093/ndt/gfl560 17005527

[33] Kruse AE Eisenberger U Frey FJ Mohaupt MG . The calcimimetic cinacalcet normalizes serum calcium in renal transplant patients with persistent hyperparathyroidism. Nephrol. Dial. Transplant. (2005) 20:1311–4. doi: 10.1093/ndt/gfh924 15941846

[34] Frey S Goronflot T Kerleau C Gourraud P-A Caillard C Hourmant M . Parathyroidectomy or cinacalcet: Do we still not know the best option for graft function in kidney-transplanted patients? A meta-analysis Surg. (2021) 170:727–35. doi: 10.1016/j.surg.2021.02.048 33810851

[35] Yavropoulou MP Kotsa K Gotzamani Psarrakou A Papazisi A Tranga T Ventis S . Cinacalcet in hyperparathyroidism secondary to X-linked hypophosphatemic rickets: case report and brief literature review. Hormones. (2010) 9:274–8. doi: 10.14310/horm.2002.1277 20688626

[36] Grove-Laugesen D Rejnmark L . Three-year successful cinacalcet treatment of secondary hyperparathyroidism in a patient with x-linked dominant hypophosphatemic rickets: a case report. Case Rep Endocrinol. (2014) 2014:479641. doi: 10.1155/2014/479641 24660072 PMC3934321

[37] Pires GO Vieira IO Hernandes FR Teixeira AL Oliveira IB Dominguez WV . Effects of parathyroidectomy on the biology of bone tissue in patients with chronic kidney disease and secondary hyperparathyroidism. Bone. (2019) 121:277–83. doi: 10.1016/j.bone.2019.01.029 30738215

[38] Charhon SA Berland YF Olmer MJ Delawari E Traeger J Meunier PJ . Effects of parathyroidectomy on bone formation and mineralization in hemodialyzed patients. Kidney Int. (1985) 27:426–35. doi: 10.1038/ki.1985.27 2581010

[39] Latus J Roesel M Fritz P Braun N Ulmer C Steurer W . Incidence of and risk factors for hungry bone syndrome in 84 patients with secondary hyperparathyroidism. Int J Nephrol. Renovasc. Dis. (2013) 6:131–7. doi: 10.2147/IJNRD.S47179 PMC3709645 23882155

[40] Ho L-Y Wong P-N Sin H-K Wong Y-Y Lo K-C Chan S-F . Risk factors and clinical course of hungry bone syndrome after total parathyroidectomy in dialysis patients with secondary hyperparathyroidism. BMC Nephrol. (2017) 18:12. doi: 10.1186/s12882-016-0421-5 28073343 PMC5223390

[41] Ramesh S Vekaria S Fisher JC Wright K Underwood H Prescott J . A novel risk score to predict hungry bone syndrome after parathyroidectomy for renal hyperparathyroidism. Endocr. Pract. (2023) 29:890–6. doi: 10.1016/j.eprac.2023.08.007 37678470

[42] Amjad W Ginzberg SP Passman JE Heintz J Kelz RR Wachtel H . Predictive risk score for post-parathyroidectomy hungry bone syndrome in patients with secondary hyperparathyroidism. J Clin Endocrinol Metab. (2023). doi: 10.1210/clinem/dgad636 37897423

[43] Boyle IT Fogelman I Boyce B Thomson JE Beastall GH McIntosh WB . 1alpha-hydroxyvitamin D3 in primary hyperparathyroidism. Clin Endocrinol 7 Suppl. (1977), 215s–22s. doi: 10.1111/j.1365-2265.1977.tb03384.x 606419

[44] Heath DA Van’t Hoff W Barnes AD Gray JG . Value of 1-alpha-hydroxy vitamin D3 in treatment of primary hyperparathyroidism before parathyroidectomy. Br Med J. (1979) 1:450–2. doi: 10.1136/bmj.1.6161.450 PMC1597771 427402

[45] Mayilvaganan S Vijaya Sarathi HA Shivaprasad C . Preoperative zoledronic acid therapy prevent hungry bone syndrome in patients with primary hyperparathyroidism. Indian J Endocrinol Metab. (2017) 21:76–9. doi: 10.4103/2230-8210.196023 PMC5240085 28217502

[46] Lee I-T Sheu WH-H Tu S-T Kuo S-W Pei D . Bisphosphonate pretreatment attenuates hungry bone syndrome postoperatively in subjects with primary hyperparathyroidism. J Bone Miner. Metab. (2006) 24:255–8. doi: 10.1007/s00774-005-0680-x 16622740

[47] Al-Jawad M Rashid AK Narayan KA . Primary hyperparathyroidism in Saudi Arabia: a review of 46 cases. Med J Malaysia. (2007) 62:282–5. 18551929

[48] Graal MB Wolffenbuttel BH . Consequences of long-term hyperparathyroidism. Neth. J Med. (1998) 53:37–42. doi: 10.1016/s0300-2977(98)00010-2 9718941

[49] Corsello SM Paragliola RM Locantore P Ingraudo F Ricciato MP Rota CA . Post-surgery severe hypocalcemia in primary hyperparathyroidism preoperatively treated with zoledronic acid. Hormones. (2010) 9:338–42. doi: 10.14310/horm.2002.1286 21112866

[50] Yong TY Li JYZ . Mediastinal parathyroid carcinoma presenting with severe skeletal manifestations. J Bone Miner. Metab. (2010) 28:591–4. doi: 10.1007/s00774-010-0173-4 20237944

[51] Pal R Gautam A Bhadada SK . Role of bisphosphonates in the prevention of postoperative hungry bone syndrome in primary hyperparathyroidism: A meta-analysis and need for randomized controlled trials. Drug Res. (2021) 71:108–9. doi: 10.1055/a-1325-0351 33296924

[52] Coco M Glicklich D Faugere MC Burris L Bognar I Durkin P . Prevention of bone loss in renal transplant recipients: a prospective, randomized trial of intravenous pamidronate. J Am Soc Nephrol. (2003) 14:2669–76. doi: 10.1097/01.asn.0000087092.53894.80 14514747

[53] Lamb YN . Burosumab: first global approval. Drugs. (2018) 78:707–14. doi: 10.1007/s40265-018-0905-7 29679282

[54] Weber TJ Imel EA Carpenter TO Peacock M Portale AA Hetzer J . Long-term burosumab administration is safe and effective in adults with X-linked hypophosphatemia. J Clin Endocrinol Metab. (2022) 108:155–65. doi: 10.1210/clinem/dgac518 PMC9759172 36072994

[55] Lafage-Proust M-H . What are the benefits of the anti-FGF23 antibody burosumab on the manifestations of X-linked hypophosphatemia in adults in comparison with conventional therapy? A review. Ther Adv Rare Dis. (2022) 3:26330040221074702. doi: 10.1177/26330040221074702 37180412 PMC10032432

[56] Ruppe MD Zhang X Imel EA Weber TJ Klausner MA Ito T . Effect of four monthly doses of a human monoclonal anti-FGF23 antibody (KRN23) on quality of life in X-linked hypophosphatemia. Bone Rep. (2016) 5:158–62. doi: 10.1016/j.bonr.2016.05.004 PMC4926842 28326356

